# Research on Benggang identification and deformation monitoring based on optical and Radar data

**DOI:** 10.1371/journal.pone.0354123

**Published:** 2026-07-27

**Authors:** Hao Liu, Hongtao Jiang, Tianyi Song, Sanxiong Chen, Chengrui Fei, Shaoqiang Huang, Anqi Zhang

**Affiliations:** 1 College of resources and environment, ZhongKai University of Agriculture and Engineering, Guangzhou, China; 2 College of horticulture and landscape architecture, ZhongKai University of Agriculture and Engineering, Guangzhou, China; ICIMOD: International Centre for Integrated Mountain Development, NEPAL

## Abstract

Benggang erosion, a severe soil erosion landform in southern China, threatens ecological security and regional development. Conventional methods can delineate surface morphology but lack capability to characterize vertical dynamics. To address it, this study presents an integrated method of deep learning and multi-temporal InSAR based on optical and radar data, and conducts benggang identification and surface deformation monitoring in typical benggang regions of Wuhua County, Guangdong, China. The results show that a U-Net model trained on Gaofen-2 high-resolution imagery achieved accurate automated Benggang delineation with a mean IoU of 86%. Subsequently, 90 Sentinel-1 SAR scenes acquired between 2022 and 2024 were processed using SBAS-InSAR and D-InSAR techniques, with Kriging interpolation employed to generate a spatially continuous deformation field. The framework successfully resolved millimeter-scale interannual vertical surface deformation, with internal cross-validation metrics (R^2^ = 0.978, RMSE = 0.544 mm, MAE = 0.313 mm) confirming high spatial consistency of the fused deformation field. The proposed framework offers a reliable and scalable technical pathway for stereoscopic Benggang monitoring and demonstrates strong potential for geohazard early-warning and ecological risk management.

## Introduction

Benggang is a distinctive soil erosion landform widely distributed in southern China, driven mainly by the combined effects of gravity and hydraulic forces that cause soil detachment, collapse, and accumulation, posing substantial threats to ecosystems, agricultural productivity, and infrastructure [1]. During long-term evolution, Benggang may transition toward landslides, with shallow erosion potentially developing into large-scale slope failures and thus increasing regional hazard risks [2]. Guangdong Province exhibits the most severe Benggang erosion, with more than 100,000 sites covering over 1,100 km^2^, accounting for about 49% of the affected area and about 74% of the total number of sites in southern China [3]. Although Benggang represents only about 8% of the national soil erosion area, it contributes nearly 43% of the sediment yield, making it one of the major drivers of land degradation and ecological deterioration. However, current research has largely focused on qualitative descriptions of morphology, mechanisms, and triggering factors [4], while intelligent recognition, dynamic monitoring, and spatiotemporal evolution analysis remain insufficiently explored [5]. Closing these gaps requires first achieving accurate boundary delineation and spatial localization of Benggang clusters, and then conducting deformation monitoring to support erosion-risk assessment and ecological hazard mitigation [6,7].

In recent years, CNN-based Deep learning has advanced rapidly in remote sensing image analysis [8]. Unlike traditional machine learning approaches such as support vector machines and decision trees, which rely heavily on handcrafted features [9,10], Deep learning enables end-to-end modeling that automatically extracts multi-level representations and captures complex spatial patterns [11,12]. Among various Deep learning architectures, the U-Net model, with its symmetric encoder–decoder structure and skip connections, has been widely adopted in medical image segmentation and has demonstrated outstanding performance in tasks such as tissue recognition and lesion detection [13,14]. More recently, U-Net has been introduced into the field of remote sensing, where it has shown promising results in applications including building extraction and land cover classification [15–17]. Introducing U-Net for Benggang recognition is therefore a novel attempt, enabling improved delineation in complex terrain by jointly leveraging contextual cues and fine spatial details, and offering a new pathway for monitoring and interpreting soil erosion processes.

Interferometric Synthetic Aperture Radar (InSAR) is an advanced remote sensing technique capable of precisely measuring subtle ground deformations [18,19]. Its core principle involves analyzing phase differences between pairs of synthetic aperture radar (SAR) images, combined with digital elevation model (DEM) data, to retrieve high-accuracy vertical and horizontal displacement information [20]. InSAR offers non-contact, all-weather, and day-and-night monitoring capabilities, enabling large-scale acquisition of surface deformation data [21,22]. Compared with traditional methods such as the Global Navigation Satellite System (GNSS), leveling surveys, GPS, and crack meters, InSAR provides significant advantages in terms of lower cost, wide spatial coverage, and high spatiotemporal resolution. These strengths have made it an increasingly important tool for ground deformation monitoring [23]. Its applications now span a wide range of fields, including co-seismic and post-seismic deformation [24], volcanic activity [25,26], landslide monitoring [27], mining subsidence [28], and urban surface deformation [29], demonstrating excellent applicability and reliability.

In complex terrain and geological settings, long-term InSAR monitoring is often challenged by temporal decorrelation, atmospheric delays, and data gaps in low-coherence areas, which can compromise the continuity and stability of deformation fields [30,31]. Different InSAR strategies exhibit complementary strengths: D-InSAR is more sensitive to short-term deformation, whereas SBAS-InSAR improves temporal continuity for long-term monitoring, thereby enhancing overall robustness when used jointly [21,31].For instance, in eco-mining areas, D-InSAR has successfully captured subtle surface deformations of mining roads induced by extraction activities [32], providing critical data support for mine restoration [33]. Moreover, D-InSAR has played an essential role in monitoring natural hazards such as earthquakes [33] and volcanic eruptions [21], offering valuable technical support for risk assessment and early warning. Nevertheless, D-InSAR performance degrades when acquisition intervals are long or vegetation changes are strong, which intensifies temporal decorrelation and noise [34]. By selecting small-baseline interferometric pairs and applying singular value decomposition (SVD), SBAS-InSAR mitigates decorrelation effects and enables more reliable time-series deformation retrieval, with successful applications in mining- and landslide-related subsidence monitoring and post-processing [21,35–37], including tracking long-term residual subsidence over 24 years in southern Alsace, France [38]. SBAS-InSAR has also supported early warning and engineering decision-making, and its effectiveness has been validated through comparisons with GNSS-based analyses in medium- to high-deformation areas [37,39,40]. Nevertheless, SBAS-InSAR remains constrained in densely vegetated areas and regions with weak surface structures, where coherence loss limits accuracy and applicability [41].

To address these critical limitations, the paper proposes an integrated framework that couples Deep learning-based Benggang recognition with InSAR deformation analysis: U-Net is applied to high-resolution imagery to delineate Benggang boundaries, followed by SBAS-InSAR and D-InSAR retrieval of cumulative deformation and annual subsidence rates within the extracted regions; Kriging-based fusion and cross-validation are then employed to optimize spatial consistency and evaluate fitting accuracy. By linking Benggang boundaries with deformation layers, the method enables dynamic identification of evolutionary trends in key erosion zones and offers early-warning insights into potential landslide risks. Meanwhile, this integration alleviates coherence-related limitations and improves the continuity, accuracy, and interpretability of deformation estimates, providing technical support for understanding Benggang evolution and its potential transition toward large-scale landslide hazards.

### Research area and data source

#### Research area.

**Fig 1:**
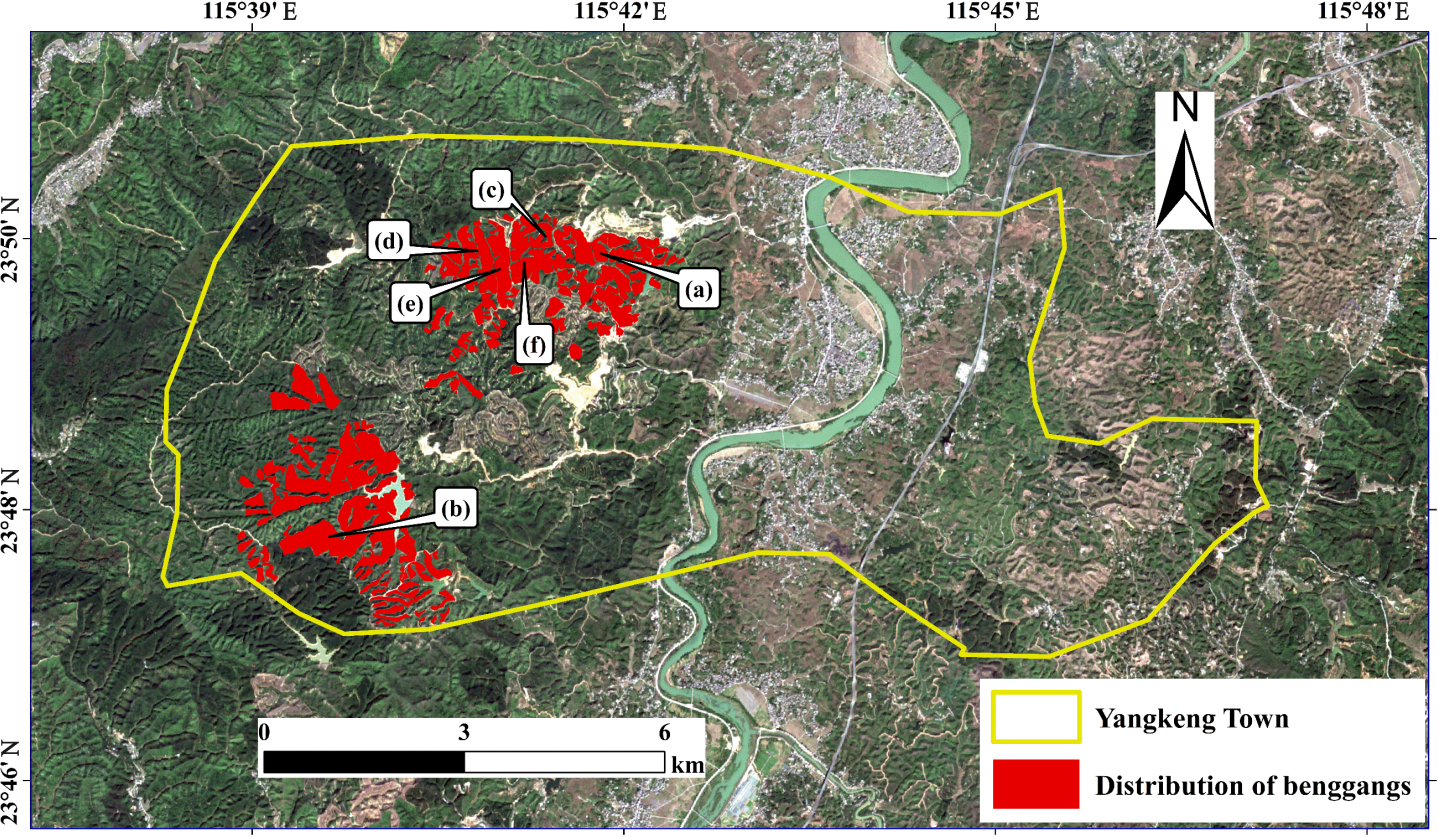
Location of the study area in Yangkeng Town, Wuhua County, Guangdong Province, China. Labeled points (a)-(h) correspond to field investigation sites (Base map: Copernicus Sentinel-2 L2A true-color image, processed by the authors.).

The study area is located in Yangkeng Town, Wuhua County, Meizhou City, Guangdong Province, China (Fig 1). It experiences a humid subtropical monsoon climate, with a mean annual temperature of about 21 °C and mean annual precipitation of about 1,500 mm, of which nearly 75% falls between April and September, producing highly seasonal and intense rainfall that drives severe erosion processes [42].

The Benggang in Yangkeng Town, Wuhua County, is strongly influenced by summer climatic conditions such as high temperatures and intense rainfall. Elevated temperatures accelerate soil moisture evaporation, causing surface layers to crack, loosen, and lose structural stability, thereby creating favorable physical conditions for Benggang initiation and development [43]. Prolonged heat also enhances weathering processes, making granite debris more susceptible to erosion and transport [44]. Intense rainfall directly triggers Benggang activity, as concentrated precipitation generates surface runoff that scours gullies, transports large volumes of sediment, and deposits it in river channels, raising riverbeds and increasing flood risk [45]. Infiltration during rainstorms further penetrates into deeper soil layers, increasing driving forces and inducing landslide-type Benggang [46]. The combined effects of heat and rainfall not only destroy farmland and degrade ecosystems but also threaten the safety of nearby settlements, while sediment transport may contaminate downstream water sources, affecting irrigation and drinking water security.

Field investigations and data collection in Yangkeng Town, Wuhua County were complied with all relevant local regulations. No specific permits were required because the work involved non-invasive observations and remote sensing in non-protected areas. In addition, UAV imagery was obtained in collaboration with the Guangdong Provincial Water Resources and Hydropower Survey and Design Institute Co., Ltd.

**Fig 2:**
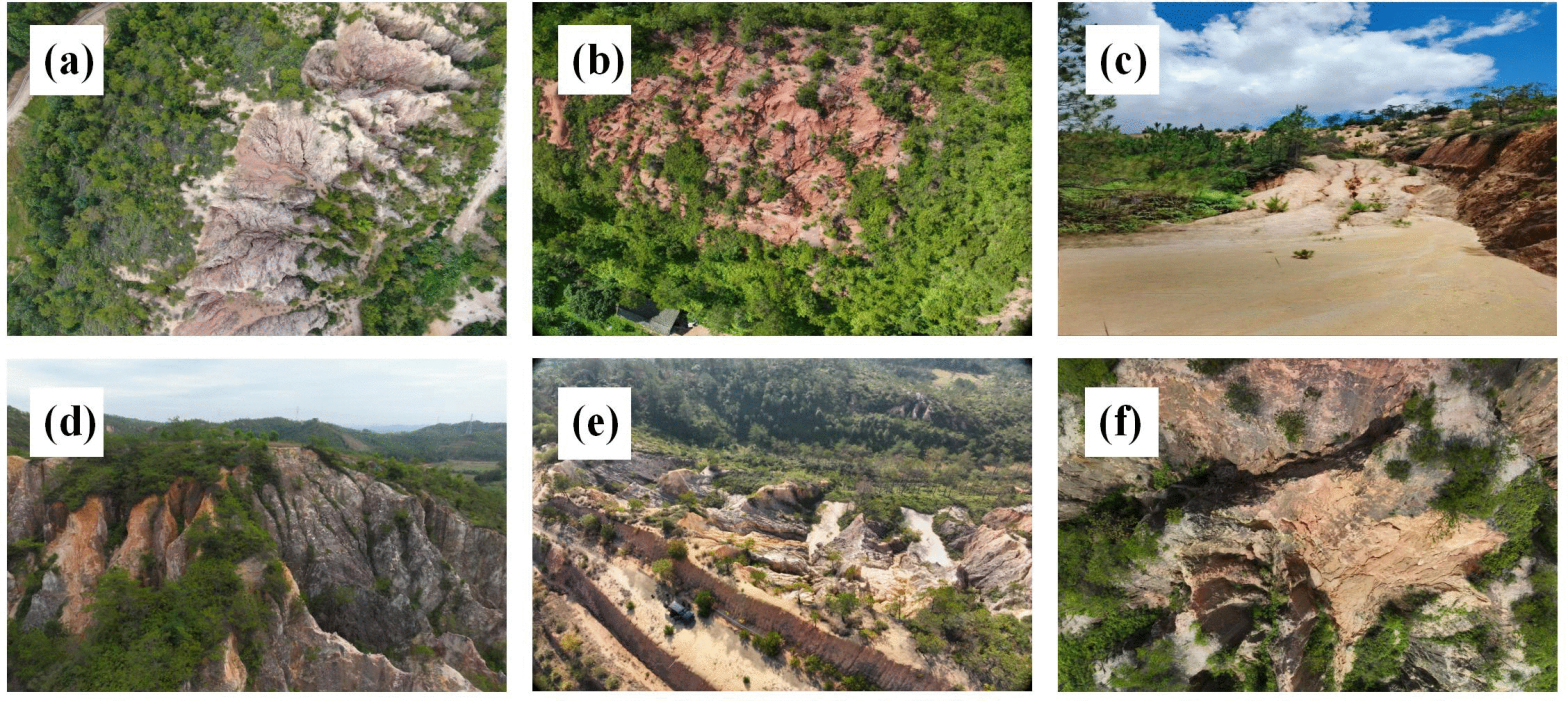
Field investigation of Benggang erosion. (a) Converging gully system; (b) Exposed soil with low vegetation; (c) Sediment deposition zone; (d) Steep scarp profile; (e) Inter-gully vegetation; (f) Collapse evidence at gully bed. (Photographs by authors using DJI UAV-3).

To assess the degree of on-site erosion, field investigations were conducted in Yangkeng Town using DJI UAV-3 aerial photography. The Benggang development captured in the images is shown in Fig 2. Fig 2a illustrates an umbrella-shaped gully system converging downslope, highlighting the dominant role of surface runoff in gully expansion. Fig 2b depicts an area with low vegetation cover and widespread exposure of lateritic red soil, reflecting weak soil resistance and an intense erosion stage. Fig 2c shows the depositional zone at the gully bottom, where sediments and bare ground alternate, indicating the cyclic processes of gully erosion and sedimentation. Fig 2d presents a typical Benggang scarp profile with steep slopes and sparse vegetation, revealing slope instability under lateral erosion. Fig 2e captures a relatively well-vegetated inter-gully area, where plant cover helps stabilize slopes and slow gully expansion. Fig 2f shows clear evidence of incision and collapse at the downstream gully bed, with visible slump deposits, suggesting the area remains in an active developmental stage. Collectively, Figs 2a–f provide intuitive evidence that Benggang in the study area simultaneously exhibits spatial patterns of erosion expansion and localized ecological recovery. To further capture these dynamics, the monitoring area was delineated in the northwestern sector of the town, where Benggang are more numerous, highly exposed, and spatially concentrated, thereby facilitating subsequent field investigations.

### Optical data

The optical imagery used in this study was acquired in 2022 by the Sentinel-2 satellite over Wuhua County, Guangdong Province, China. A cloud-free Sentinel-2 scene was selected to ensure stable radiometric conditions and to facilitate extraction of terrain-related texture features, providing a reliable basis for Benggang mapping and subsequent analysis. In addition, Gaofen-2 high-resolution imagery was used to develop and train the U-Net deep learning model.

### SAR data

The SAR data were obtained from the European Space Agency’s Sentinel-1A satellite in interferometric wide-swath (IW) mode, with single look complex (SLC) products. The study area was covered by C-band VV-polarized acquisitions collected between January 6, 2022, and December 31, 2024, resulting in a total of 90 SAR scenes. The satellite operated with a 12-day repeat cycle, and all data were from track number 113. During processing, precise orbit files corresponding to the SAR acquisition period were used to eliminate orbital errors. The Shuttle Radar Topography Mission (SRTM) DEM with 30 m resolution, jointly released by NASA and the National Imagery and Mapping Agency (NIMA), was applied for topographic phase removal and geocoding.

### Research methods

**Fig 3:**
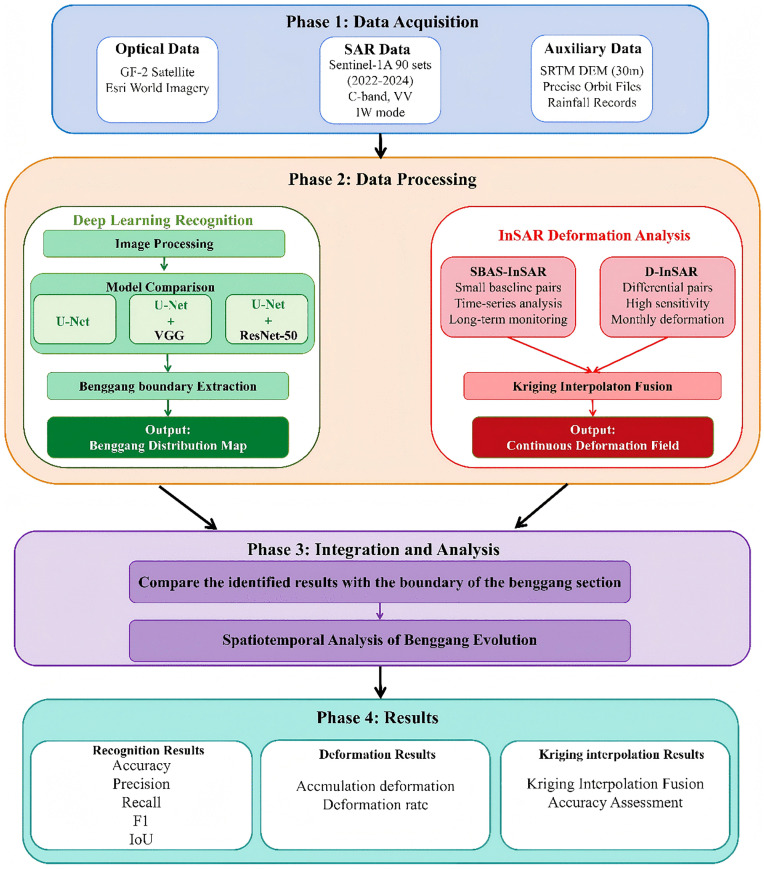
Overall workflow diagram.

The research methodology consisted of two main components. First, a Deep learning model was used to delineate Benggang boundaries, particularly suited for large-scale Benggang clusters. Second, the identified areas were monitored using SBAS-InSAR and D-InSAR techniques to retrieve cumulative deformation and deformation rates, thereby characterizing the temporal evolution of Benggang within the study period (Fig 3).

### U-Net Deep learning model

**Fig 4:**
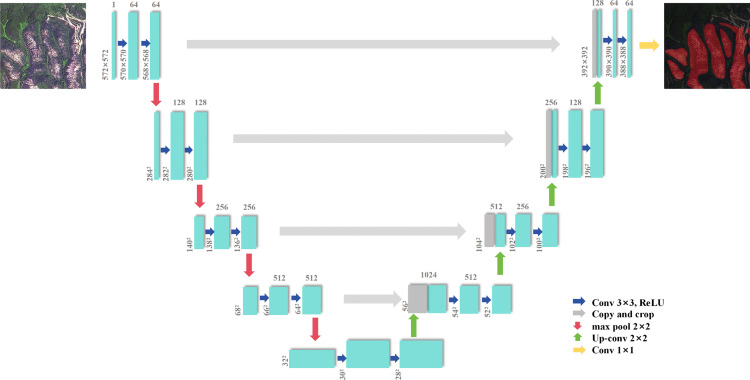
Architecture of the U-Net model (The example background is a cropped Gaofen-2 high resolution satellite image obtained via the Guangdong Province Gaofen Remote Sensing Data Management Platform under institutional authorization.).

The Deep learning framework adopted in this study was the U-Net architecture (Fig 4), which is a fully convolutional encoder–decoder network originally designed for pixel-level semantic segmentation tasks. U-Net is characterized by its symmetric contracting and expanding paths, coupled with skip connections that directly fuse low-level spatial details from the encoder with high-level semantic features in the decoder. This architecture enables precise boundary delineation and robust feature localization, making it particularly suitable for extracting geomorphic targets with complex shapes and sharp elevation transitions, such as Benggang landforms. The U-Net framework comprises three key modules: the backbone feature extraction module, the feature enhancement module, and the prediction module. In the first stage, the backbone feature extraction module utilizes stacked convolutional and max-pooling layers to progressively derive abstract semantic features from the input imagery. Two backbone networks were tested: VGG [47] and ResNet-50 [48]. VGG is characterized by its simple and uniform structure, making it suitable for transfer learning, while ResNet-50 incorporates residual connections to effectively mitigate gradient vanishing, enabling stable training of deeper networks. Both networks provide strong feature extraction capabilities and are well suited for complex image recognition tasks; thus, they were selected for Benggang recognition. Each convolutional layer employed a 3 × 3 kernel followed by a ReLU activation function, which enhances non-linear feature representation and facilitates the capture of fine image details.

The convolution operation formula is:


$$\begin{array}{c} \text{Z }=\text{ W }*\text{ X }+\text{ b} \end{array}$$ (1)

W is the convolution kernel, X is the input feature map, b is the bias term, and * denotes the convolution operation.

The second stage corresponds to the feature enhancement and decoding module. In this stage, skip connections are employed to fuse high-level semantic features obtained through upsampling with the corresponding low-level detail features from the encoder, thereby preserving spatial accuracy while enhancing semantic resolution. Each decoding unit consists of an upsampling layer, a feature concatenation operation, and two successive 3 × 3 convolutional layers followed by ReLU activation functions, forming the characteristic U-shaped architecture.

The feature fusion formula for skip connections is:


$$\begin{array}{c} {\text{F}}_{\text{skip}}\;=\text{ Concat}({\text{F}}_{\text{encoder}},\;{\text{F}}_{\text{decoder}}) \end{array}$$ (2)

${\text{F}}_{\text{encoder}}\;$ and ${\text{F}}_{\text{decoder}}$ respectively denote the feature maps of the corresponding layers in the encoder and decoder [49].

The third stage is the prediction module, which integrates the multi-scale features extracted in the previous stages to generate the final segmentation map. This is achieved by applying a 1 × 1 convolutional layer to map the feature representations to the target number of classes, followed by a Sigmoid activation function to produce pixel-wise classification probabilities.

The sigmoid activation function is used to map the output to between 0 and 1, which is used to generate the resolution probability of each pixel. The sigmoid activation function formula:


$$\begin{array}{c} \sigma (\text{x})\;=\;\frac{\text{1}}{\text{1}+{\text{e}}^{-\text{x}}} \end{array}$$ (3)

### SBAS-InSAR and D-InSAR techniques

SBAS-InSAR technique is one of the key branches of time-series InSAR analysis. In this study, a total of 90 Sentinel-1 SAR images were acquired to ensure sufficient temporal coverage of the study area. Based on the spatial distribution and temporal intervals of the data, appropriate temporal and spatial baseline thresholds were defined to construct interferometric pairs that satisfy the small-baseline principle, thereby minimizing spatial and temporal decorrelation effects [50]. For each interferometric pair, differential interferometric processing was first performed to extract unwrapped phase information. Temporal high-pass and spatial low-pass filtering strategies were then applied to effectively mitigate atmospheric delay effects. Finally, time-series deformation estimates were derived for each pixel using a least-squares inversion [51], yielding both the temporal evolution of surface deformation and the average deformation rate across the study area. A spatially stable reference point was automatically selected by the SARscape SBAS module based on maximum temporal coherence criteria. The reference point is located on exposed bedrock in the eastern portion of the study area, outside the active erosion zone. All reported deformation values represent relative line-of-sight displacements with respect to this reference.


$$\begin{array}{c} \Phi_{\text{x},\text{y}}^{\text{k}}=\varphi_{\text{top},\text{x},\text{y}}^{\text{k}}+\varphi_{\text{def},\text{x},\text{y}}^{\text{k}}+\varphi_{\text{orb},\text{x},\text{y}}^{\text{k}}+\varphi_{\text{atm},\text{x},\text{y}}^{\text{k}}+\varphi_{\text{noi},\text{x},\text{y}}^{\text{k}} \end{array}$$ (4)

$$\begin{array}{c} \varphi_{\text{def},\text{x},\text{y}}^{\text{k}}=-\frac{\text{4}\pi }{\lambda }\Delta {\text{r}}_{\text{x},\text{y}}^{\text{k}}=-\frac{\text{4}\pi }{\lambda }\sum_{\text{j}={\text{S}}_{\text{k}+\text{1}}}^{{\text{M}}_{\text{k}}}\left({\text{t}}_{\text{j}}-{\text{t}}_{\text{j}-\text{1}}\right){\text{v}}_{\text{j}} \end{array}$$ (5)

$$\begin{array}{c} \varphi_{\text{top},\text{x},\text{y}}^{\text{k}}=-\frac{\text{4}\pi }{\lambda }\frac{{\text{B}}_{⊥,\text{x},\text{y}}^{\text{k}}}{{\text{r}}_{\text{x},\text{y}}^{\text{k}}\text{sin}\theta_{\text{x},\text{y}}^{\text{k}}}\Delta {\text{h}}_{\text{x},\text{y}} \end{array}$$ (6)

In Eq. (*), $\Phi_{\text{x},\text{y}}^{\text{k}}$ denotes the total interferometric phase at pixel (x,y) in the k-th interferogram; $\varphi_{\text{top},\text{x},\text{y}}^{\text{k}}$ represents the topographic phase, which can be removed using a digital elevation model (DEM); $\varphi_{\text{def},\text{x},\text{y}}^{\text{k}}$ is the deformation phase, indicating the ground displacement between interferometric acquisitions; $\varphi_{\text{orb},\text{x},\text{y}}^{\text{k}}$ is the orbital error phase, which can be corrected using precise orbital data; $\varphi_{\text{atm},\text{x},\text{y}}^{\text{k}}$ corresponds to atmospheric delay effects, typically caused by spatiotemporal variations in tropospheric refraction; and $\varphi_{\text{noi},\text{x},\text{y}}^{\text{k}}$ is noise-related phase, which is an unavoidable error source in data processing [52].

$\Delta {\text{r}}_{\text{x},\text{y}}^{\text{k}}$is the slant-range deformation of pixel (x,y) in the k-th interferogram; ${\text{t}}_{\text{j}}$ denotes the j-th acquisition time; ${\text{v}}_{\text{j}}$ is the deformation velocity of pixel (x,y) along the radar line of sight during the j-th time interval; ${\text{B}}_{⊥,\text{x},\text{y}}^{\text{k}}$ is the perpendicular baseline at pixel (x,y)for the k-th interferogram; ${\text{r}}_{\text{x},\text{y}}^{\text{k}}$ is the slant range between the sensor and pixel (x,y); $\theta_{\text{x},\text{y}}^{\text{k}}$ is the local incidence angle; and $\Delta {\text{h}}_{\text{x},\text{y}}$ denotes the DEM error relative to the reference DEM. Finally, singular value decomposition (SVD) is applied to integrate the deformation information at each observation point, yielding the overall deformation field across the study area.

D-InSAR is essentially an extension of conventional InSAR, achieved through secondary differencing of interferograms. By incorporating a high-precision digital elevation model (DEM), D-InSAR effectively removes topographic and atmospheric phase components, thereby enabling the retrieval of high-accuracy surface deformation information. In this study, 30 Sentinel-1 SAR scenes (Path 113) acquired between January and December 2024 were processed to cover the entire Wuhua County. The 30 SLC images were processed using ENVI software, where DEM data were introduced and interferometric pairs were generated under the same polarization mode. Differential interferograms were constructed between consecutive acquisitions to produce displacement maps.

Noise was suppressed and signal quality enhanced using filtering algorithms, while coherence maps were simultaneously computed to assess the quality of interferometric pairs. Phase unwrapping was performed using the minimum cost flow algorithm to recover continuous phase information [53]. During orbit refinement and re-flattening, ground control points (GCPs) were selected based on three criteria: (1) consistently high coherence across the majority of interferometric pairs, (2) location on geologically stable terrain (bedrock outcrops) with no evidence of active deformation, and (3) successful phase unwrapping without residual fringes. A total of six GCPs were distributed across the study area to constrain residual orbital ramps and phase offsets. The spatial distribution of GCPs ensured coverage of both near-range and far-range positions within the SAR scene to minimize geometric biases. Finally, absolute phase values were converted into line-of-sight (LOS) displacements, which were geocoded to obtain the deformation results. The least-squares estimation formula is expressed as follows [54]:


$$\begin{array}{c} \text{Cost}=\sum_{\text{i}=\text{1}}^{\text{n}}{\text{c}}_{\text{i}}\cdot {\text{d}}_{\text{i}} \end{array}$$ (7)

**Fig 5:**
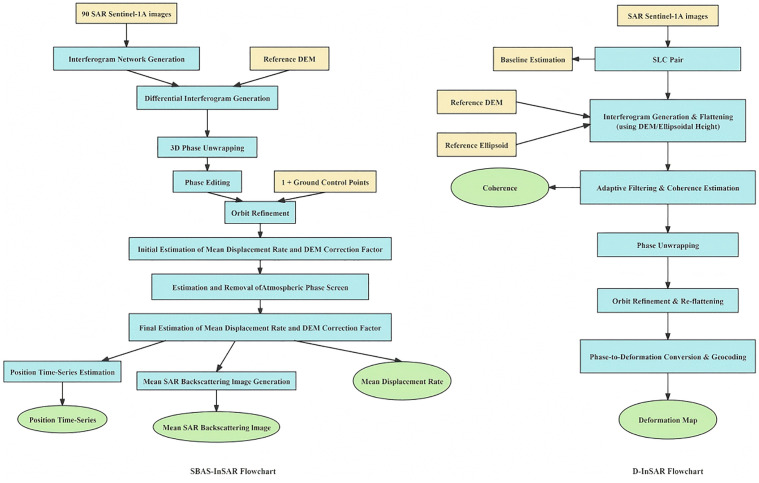
Workflow of the two InSAR techniques:SBAS-InSAR (left) and D-InSAR(right).

Cost represents the total cost, $c_{i}$ denotes the unit cost of the i-th pixel, and ${\text{d}}_{\text{i}}$ refers to the distance or path length from the source point to the i-th pixel. The workflow of the two InSAR techniques is illustrated in Fig 5.

### Atmospheric Correction and Uncertainty Analysis

Systematic mitigation of atmospheric phase screen (APS) contributions was performed using the Generic Atmospheric Correction Online Service (GACOS), which derives zenith total delay (ZTD) estimates from ECMWF ERA-5 reanalysis fields through an iterative tropospheric decomposition model. Corrections were applied to all 318 interferograms throughout the monitoring period, reducing the spatially correlated phase standard deviation from 8.77 mm to 5.64 mm and confirming effective suppression of large-scale tropospheric signals. The calculation process for the GACOS atmospheric phase:


$$\begin{array}{c} \varphi_{GACOS}^{k}(x,y)=\frac{\text{4}\pi }{\lambda }\cdot \left[{\text{ZTD}}^{\text{k}}(\text{x},\text{y})-{\text{ZTD}}^{\text{m}}(\text{x},\text{y})\right]\cdot \frac{\text{1}}{\text{cos}\theta } \end{array}$$ (8)

${\text{ZTD}}^{\text{k}}(\text{x},\text{y})$denotes the zenith total delay at location(x,y), interpolated from global atmospheric model data corresponding to the acquisition date of image k; ${\text{ZTD}}^{\text{m}}(\text{x},\text{y})$ represents the zenith total delay at location (x,y) on the acquisition date of the primary image; θ is the radar incidence angle; and $\frac{\text{1}}{\text{cos}\theta }$ is the mapping function that converts the zenith total delay to the slant range delay.

For SBAS-InSAR, residual short-wavelength turbulent delays were further attenuated through temporal high-pass and spatial low-pass filtering, improving the signal-to-noise ratio of millimeter-level deformation time series under humid subtropical conditions. D-InSAR processing was restricted to short-temporal-baseline, high-coherence interferogram pairs, with those exhibiting evident atmospheric anomalies excluded prior to analysis. Polynomial phase refinement and coherence-weighted optimization were subsequently applied to minimize residual orbital ramps, phase-unwrapping ambiguities, and vegetation-induced decorrelation. Pixels with temporal coherence below 0.3 were masked to exclude noise-dominated regions. The residual atmospheric contribution after correction was confirmed to be an order of magnitude smaller than the observed peak subsidence signal, ensuring the derived deformation field reflects actual Benggang surface dynamics.

### Kriging interpolation

In this study, Kriging interpolation was employed to integrate the results of SBAS-InSAR and D-InSAR, thereby compensating for the limitations of each individual technique in terms of spatiotemporal continuity and producing a more accurate and complete deformation field. To maximize their complementary strengths, the SBAS-InSAR results from 2024 were first supplemented and both SBAS-InSAR and D-InSAR deformation raster maps were converted into point datasets containing spatial coordinates and displacement values. Masking was then applied to retain SBAS-InSAR results in regions with high coherence, while corresponding D-InSAR values were introduced to fill gaps in areas where SBAS-InSAR failed to provide reliable measurements. The fused point dataset was subsequently interpolated using a Kriging model. During interpolation, a spherical semi-variogram was fitted to characterize the spatial autocorrelation of deformation points, resulting in a spatially continuous deformation field. This fused product preserved the spatial detail of D-InSAR while incorporating the temporal stability of SBAS-InSAR, thereby providing a more complete and reliable representation of ground subsidence patterns across the study area. The Kriging interpolation and covariance structure model are expressed as follows [55]:


$$\begin{array}{c} \hat{\text{Z}}({\text{S}}_{\text{0}})\;=\;\sum_{\text{i}=\text{1}}^{\text{N}}\lambda_{\text{i}}\text{Z}\left({\text{S}}_{\text{i}}\right) \end{array}$$ (9)

$$\begin{array}{c} \sum_{\text{j}=\text{1}}^{\text{n}}\lambda_{\text{j}}\gamma ({𝐬}_{\text{i}}-{𝐬}_{\text{j}})+\mu =\gamma ({𝐬}_{\text{i}}-{𝐬}_{\text{0}}),\;\forall \text{i}=\text{1},\ldots ,\text{n} \end{array}$$ (10)

In Equation(9), $\hat{\text{Z}}({\text{S}}_{\text{0}})$ represents the estimated value at interpolation point ${\text{S}}_{\text{0}}$；$\text{Z}\left({\text{S}}_{\text{i}}\right)$ is the true value at location ${\text{S}}_{\text{i}}$; N denotes the number of sample points; $\lambda_{\text{i}}$ is the weight assigned to the i-th sample point. These weights are calculated based on the covariance function between the sample points and the interpolation point. To ensure unbiased estimation, the weights satisfy the condition $\sum \lambda_{\text{i}}=\text{1}$. In Equation (10)，$\gamma ({𝐬}_{\text{i}}-{𝐬}_{\text{0}})$ denotes the semivariogram function, and μ is the Lagrange multiplier introduced to guarantee the constraint on the weights, ensuring that their sum equals 1.

To verify the accuracy of the interpolated results, this study employed covariance function–based Kriging interpolation to generate a deformation field with improved spatial continuity and reliability. Kriging served a dual purpose in this research: first, during the data fusion stage, it integrated the temporal continuity advantage of SBAS-InSAR with the spatial coverage advantage of D-InSAR to produce a more reasonable deformation distribution; and second, during the accuracy assessment stage, it enabled evaluation of the robustness and generalization ability of the interpolation model through cross-validation. Specifically, in the cross-validation process, each monitoring point in the fused dataset was sequentially removed, and its value was re-estimated using the remaining points via Kriging. The predicted value was then compared against the true observation. Finally, statistical indicators including RMSE, MAE, and R^2^ were calculated to quantitatively assess the accuracy of the fused results.

### Experimental results and analysis

#### Analysis of Benggang recognition results.

In this study, the U-Net semantic segmentation model demonstrated strong performance in Benggang recognition, particularly when combined with backbone feature extractors such as VGG and ResNet-50 [56]. From the prediction results (Table 1), the U-Net model showed clear advantages in areas with low vegetation cover, where it more effectively restored the spatial structure and boundary details of Benggang. This makes it especially suitable for interpreting remote sensing imagery of fragmented slopes and gully-dominated landscapes in southern China.

**Table 1:** Training and testing performance of U-Net variants for Benggang segmentation.

	Model	Accuracy	Precision	Recall	F1	IoU
Training	U-Net + VGG	82.65%	78.08%	81.10%	79.56%	67.08%
	U-Net + ResNet-50	91.99%	93.59%	90.19%	91.83%	86.54%
	U-Net	90.80%	91.20%	89.20%	90.19%	82.13%
	U-Net + VGG	91.18%	77.13%	79.55%	78.32%	67.58%
Testing	U-Net + ResNet-50	91.52%	93.44%	90.31%	91.85%	86.00%
	U-Net	91.32%	88.50%	86.00%	87.23%	77.36%

Experimental results confirmed that the ResNet-50 + U-Net model outperformed the VGG + U-Net model throughout the training process. Specifically, the VGG + U-Net achieved an overall accuracy of 82.65%, precision of 78.08%, recall of 81.10%, F1-score of 79.56%, and IoU of 67.08%. In contrast, ResNet-50 + U-Net achieved 91.99%, 93.59%, 90.19%, 91.83%, and 86.54% for the same metrics, representing improvements of 9.34% in accuracy and 15.51%, 9.09%, 12.27%, and 19.46% in precision, recall, F1-score, and IoU, respectively. As an additional baseline, the plain U-Net model exhibited intermediate performance. It achieved an accuracy of 90.80%, precision of 91.20%, recall of 89.20%, an F1-score of 90.19%, and an IoU of 82.13%. These results indicate that introducing ResNet-50 as the encoder further strengthens feature representation and boundary delineation beyond the standard U-Net architecture.

**Fig 6:**
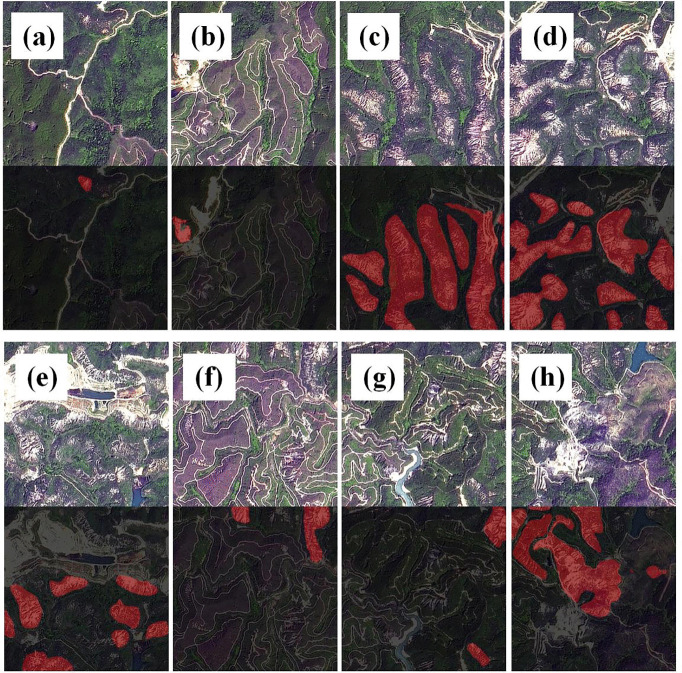
Benggang recognition results (The background is a cropped Gaofen-2 high-resolution satellite image obtained via the Guangdong Province Gaofen Remote Sensing Data Management Platform under institutional authorization. ).

The recognition performance was particularly strong in the northwestern exposed Benggang area of the study region, where accuracy and completeness reached the highest levels (As shown in Fig 6). This can be attributed to the scale and developmental characteristics of the Benggang clusters in this area. The region contains the largest and densest distribution of Benggang, forming a concentrated and contiguous spatial pattern. Long-term soil erosion has resulted in pronounced land degradation, with intersecting gullies and well-developed erosion channels that exhibit strong spectral contrasts and textural features in remote sensing imagery, thereby providing optimal conditions for recognition. Furthermore, the geological and geomorphological conditions of this area are relatively uniform, being primarily controlled by lithology, soil type, hydrology, and slope gradient. This consistency in morphological and spectral characteristics further enhances recognizability. Consequently, this region is particularly well suited for deformation monitoring and further analysis of Benggang evolution.

### Benggang deformation results

#### SBAS-InSAR for deformation monitoring.

**Fig 7:**
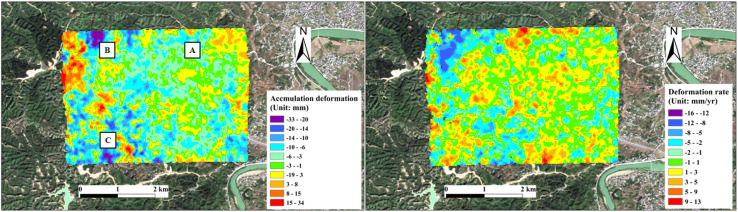
Cumulative deformation and deformation rates (Base map: Copernicus Sentinel-2 L2A true-color image, processed by the authors. ).

Fig 7 presents the Benggang deformation results along the line-of-sight (LOS) direction derived from SBAS-InSAR, where positive values indicate uplift and negative values indicate subsidence. The deformation pattern is spatially heterogeneous, with the maximum subsidence over the three-year period reaching −43 mm, primarily concentrated in the northwestern part of the Benggang area. Most subsidence values range between −15 mm and −5 mm, while several areas exceed −25 mm, mainly located near reservoirs, forest roads, and farmland margins. The maximum uplift is 43 mm, distributed mainly along roads and parts of forested areas. Uplift is predominantly within 0–10 mm, with a few localized zones exhibiting 10–25 mm of elevation gain.

Further analysis of deformation rates reveals the temporal evolution of Benggang deformation during the monitoring period. Overall, the spatial distribution of deformation rates is highly consistent with the cumulative deformation results, suggesting a strong spatial correlation between subsidence and uplift. Subsidence rates range from −16 to −1 mm/yr, with most areas exhibiting steady subsidence between −5 and −2 mm/yr, indicating a gradual but stable downward trend. Localized areas exceed −12 mm/yr, corresponding to anomalous cumulative subsidence, reflecting significant surface activity. In contrast, certain areas exhibit uplift rates of 1–13 mm/yr, which are primarily associated with terrain variability, vegetation cover, and anthropogenic activities. Taken together, the deformation rate and cumulative displacement results are mutually reinforcing, not only revealing the spatial distribution of typical Benggang but also capturing its long-term evolutionary trends, thereby providing a solid foundation for future studies on soil erosion processes and landform development.

**Fig 8:**
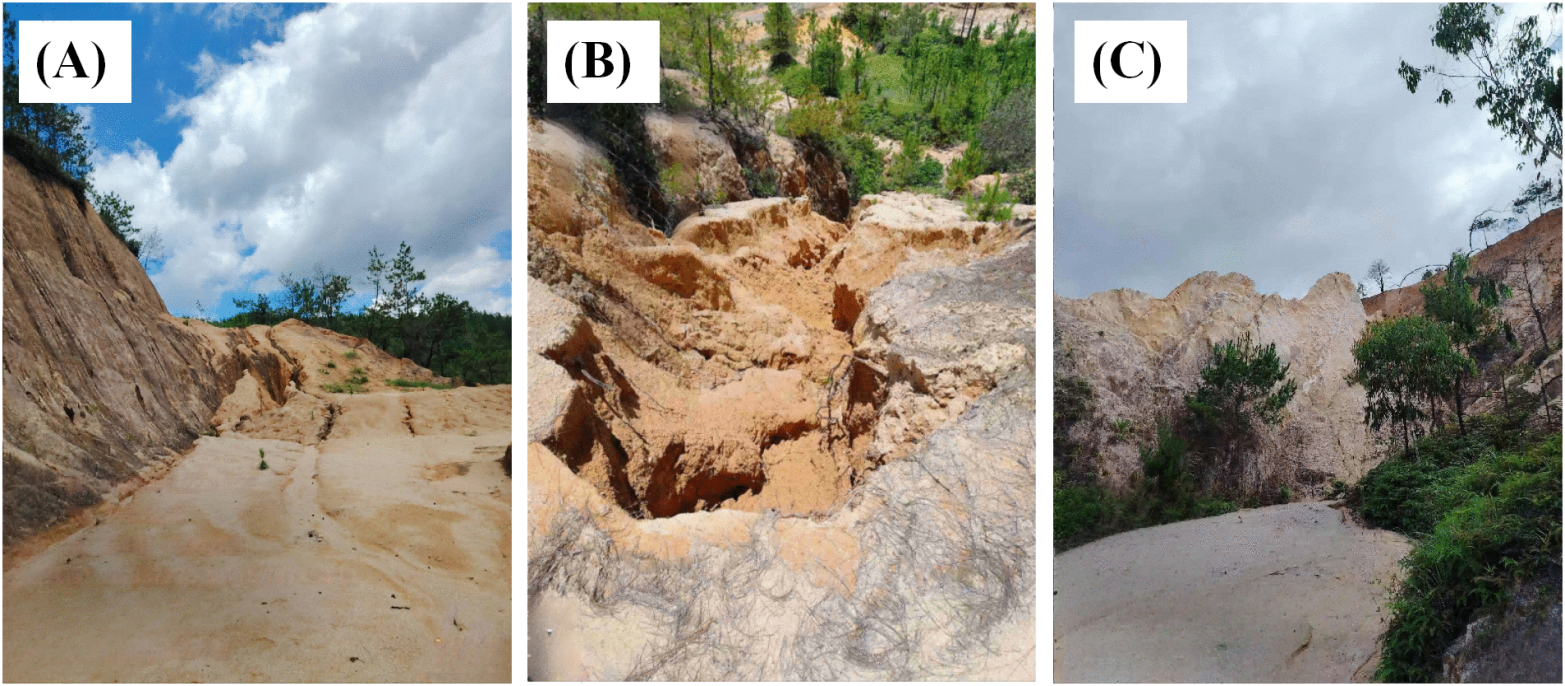
Field photographs of three representative Benggang monitoring points (A,B,C) selected for time-series deformation analysis (Photographs by authors using DJI UAV-3).

To characterize spatiotemporal deformation variability across different geomorphological settings, three representative monitoring points were selected from high-coherence pixels within the active Benggang zone: Point A at a gully head, Point B on a depositional slope, and Point C at a steep scarp (locations shown in Fig 7). These points were chosen to represent distinct erosion sub-environments rather than to serve as geodetic reference benchmarks. Their time-series deformations are expressed as relative displacements with respect to the InSAR reference point. Their corresponding positions are indicated in Fig 8.

**Fig 9:**
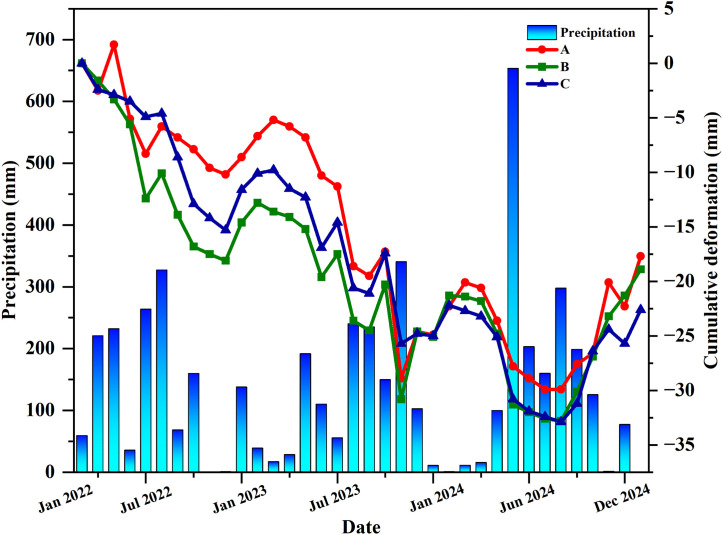
Time-series surface deformation at three representative monitoring points (A,B,C), referenced to the initial Sentinel-1 image in 2022.

As shown in Fig 9, the results reveal pronounced spatiotemporal variability and a clear response to rainfall. At Point A, cumulative deformation fluctuated between 2 mm and −30 mm, with peaks occurring in March 2022 (2 mm) and August 2024 (−30 mm), indicating the strongest sensitivity to precipitation. Point B exhibited cumulative deformation of −19 mm, with the maximum displacement of −31 mm in September 2023, showing high correlation with seasonal rainfall patterns. Point C remained within 0 to −32 mm, with the maximum deformation (−33 mm) observed in March 2024.

Rainfall data show monthly precipitation ranging from 0.2 to 653 mm. When monthly rainfall exceeded 200 mm, all three monitoring points exhibited accelerated deformation, particularly during periods of heavy rainfall in July–August 2022 and August–September 2023. Despite the complex geological structures and relatively high soil density at these sites, further observations indicate a lagged relationship between rainfall and deformation, with subsidence peaks typically occurring weeks to months after rainfall peaks. This pattern suggests a spatiotemporal process of infiltration, transmission, and progressive strength degradation.

#### D-InSAR for deformation monitoring.

**Fig 10:**
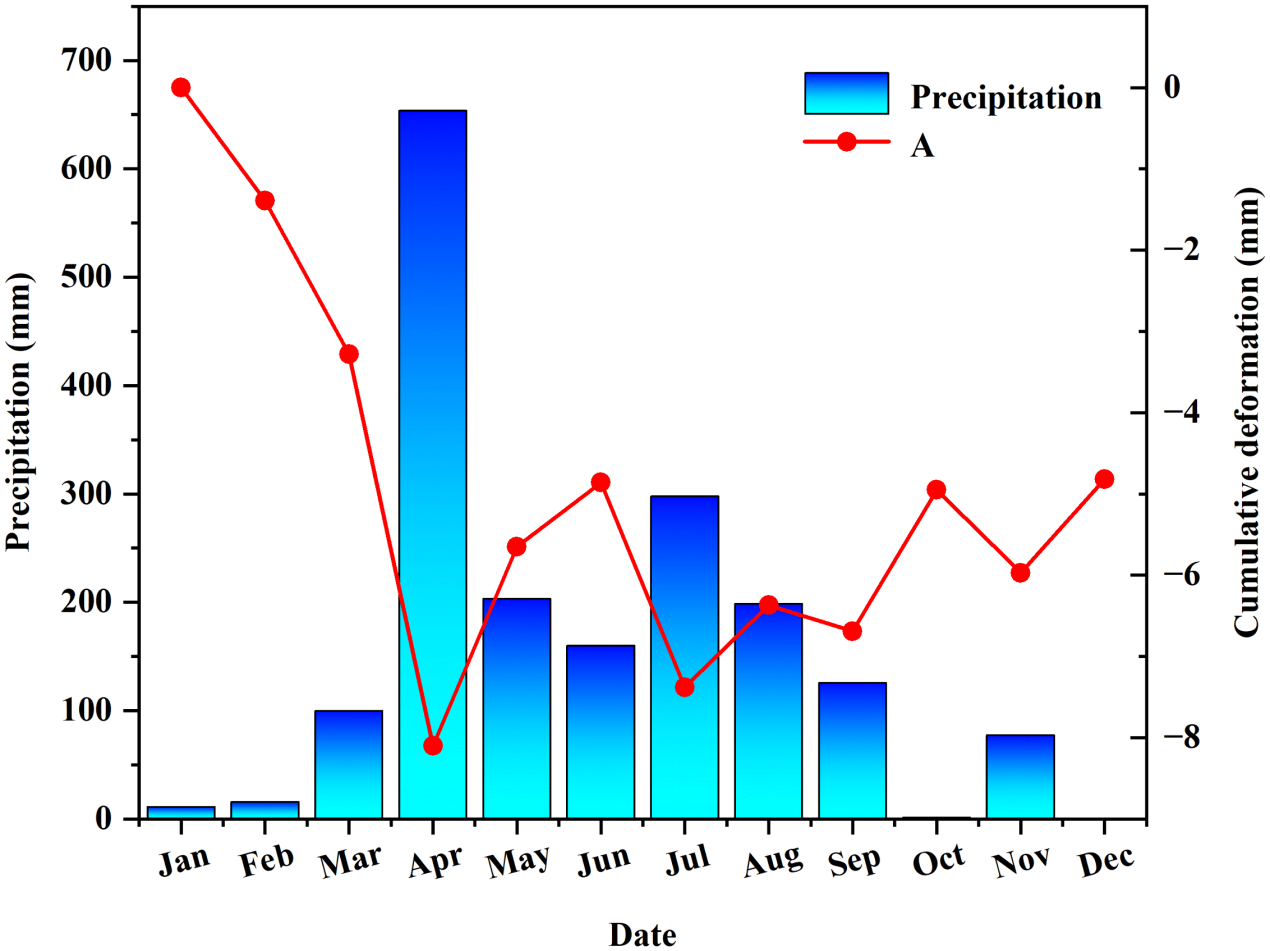
Time-series deformation derived from D-InSAR at Point A.

The D-InSAR results reveal pronounced spatiotemporal heterogeneity in surface deformation across the study area. Temporally, both the magnitude and direction of deformation varied by month: some areas experienced slight subsidence during January–March, which then intensified into significant subsidence during April–May. Spatially, deformation amplitudes and directions also exhibited considerable variability across different zones. For example, at Point A, the maximum subsidence occurred primarily in April and June–July, with values of −8.10 mm in April and −7.38 mm in June–July. These periods coincide with the local rainy season, when precipitation reaches its annual peak. The seasonal pattern of rainfall showed strong correspondence with the temporal distribution of surface subsidence, indicating that precipitation is one of the dominant factors driving deformation in the study area. The relationship between D-InSAR-derived deformation and monthly rainfall is illustrated in Fig 10.

**Fig 11:**
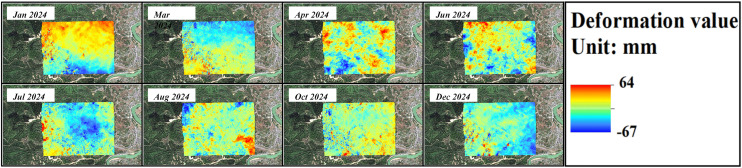
Selected monthly D-InSAR deformation maps in 2024 (Base map: Copernicus Sentinel-2 L2A true-color image processed by the authors. ).

Fig 11 presents the 2024 time-series deformation results for monitoring Point A, providing an intuitive depiction of the spatiotemporal evolution of ground deformation.

### Kriging interpolation and accuracy assessment

To quantitatively evaluate the accuracy differences between D-InSAR and SBAS-InSAR in long-term deformation monitoring, 30 Sentinel-1A scenes acquired in 2024 were analyzed. First, 29 differential interferograms were generated using the two-image differencing method, and cumulative deformation results for 2024 were obtained through sequential accumulation. Subsequently, a SBAS network was constructed from same-track, same-polarization images to derive monthly time-series deformation and annual velocity fields. Both InSAR products were reprojected to a WGS-84 ellipsoid with a spatial resolution of 30 m, and pixels with a coherence coefficient of γ ≥ 0.7 were selected as common analysis units. For each pixel, the cumulative displacement derived from 29 D-InSAR interferograms was compared against the cumulative subsidence obtained from SBAS-InSAR, and accuracy was statistically assessed using three metrics: root mean square error (RMSE), mean absolute error (MAE), and coefficient of determination (R^2^).

**Fig 12:**
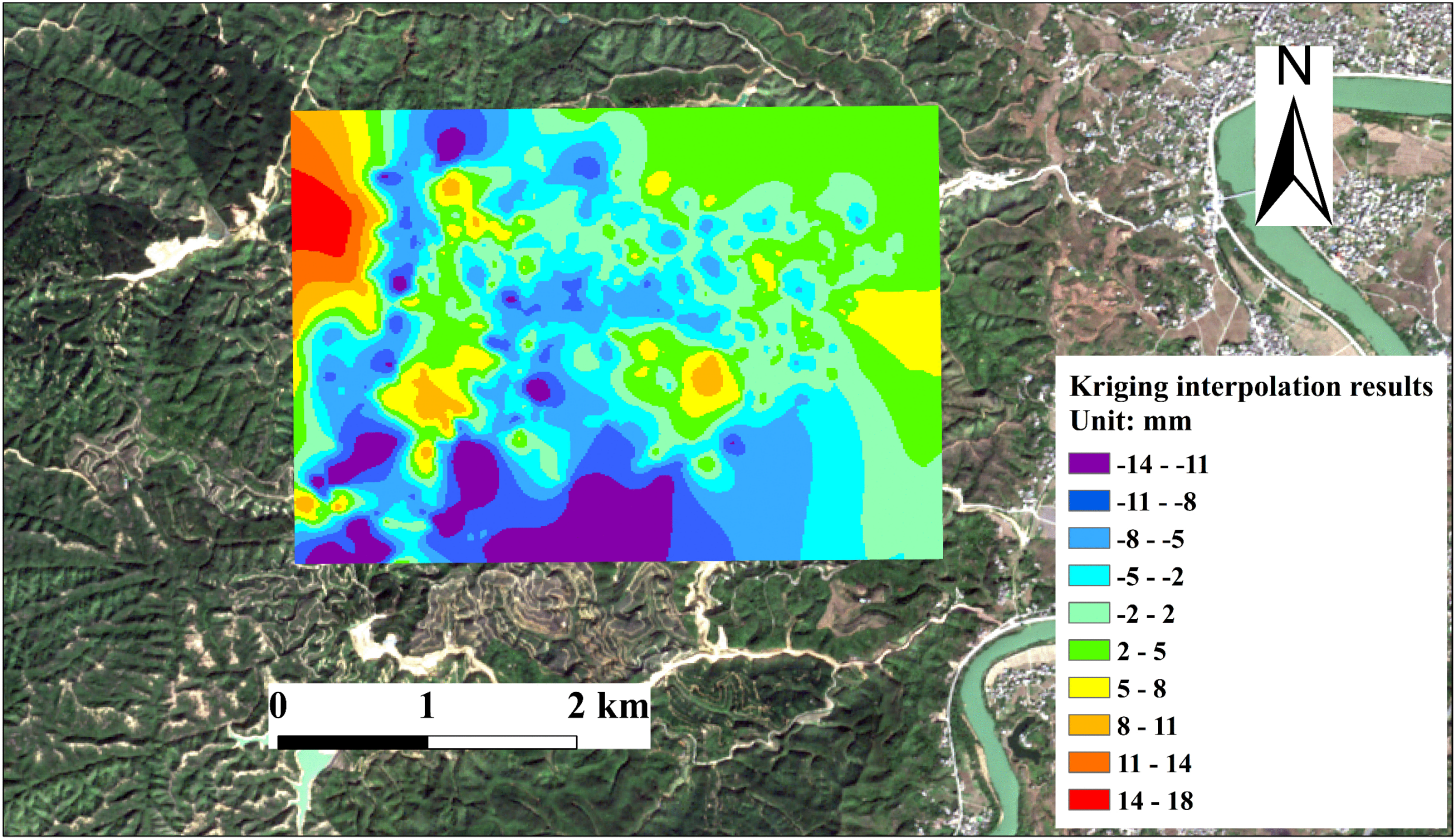
Interpolated SBAS-InSAR deformation results for 2024 (Base map: Copernicus Sentinel-2 L2A true-color image, processed by the authors. ).

The fused deformation points were further processed through Kriging interpolation to generate a continuous cumulative subsidence map for the study area. The interpolation results indicate a deformation range of −14–18 mm, with a mean value of −1.33 mm and a standard deviation of 3.65 mm. The ratio of the standard deviation to the mean is about 2.7, indicating a relatively stable performance level. Kriging interpolation significantly enhanced the accuracy and reliability of the fused InSAR results, yielding deformation magnitudes and directions more consistent with actual ground conditions. This not only highlights the spatial complexity of Benggang deformation but also ensures the rationality of the data. As illustrated in Fig 12, the optimized deformation field clearly depicts the spatial characteristics of Benggang deformation.

After applying Kriging interpolation, three standard statistical metrics were used to quantitatively evaluate the model. From an error analysis perspective, the root mean square error (RMSE) was 0.544 mm and the mean absolute error (MAE) was 0.313 mm, both at relatively low levels, indicating that the deviations between predicted and observed values were minimal. Notably, the coefficient of determination (R^2^) reached 0.978, suggesting that the model provided an excellent fit to the observations and effectively captured the spatial variability of interpolated deformation. These metrics quantify the internal consistency and interpolation performance of the fused model rather than the absolute measurement accuracy of the InSAR-derived displacements, as the latter would require independent geodetic validation.

**Fig 13:**
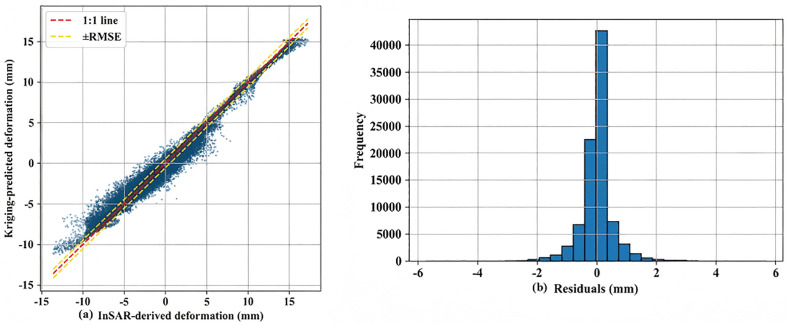
Cross-validation scatterplot and residual histogram.

Fig 13(a) presents the cross-validation scatterplot, where data points are densely clustered along the 1:1 line; more than 85% of the samples fall within ±RMSE, implying that most prediction errors were smaller than one RMSE. Outliers beyond ±RMSE were mainly concentrated in areas of low coherence or steep deformation gradients, indicating that residuals were dominated by local geological variability rather than systematic model bias. Approximately 68% of the data are distributed within the range of −5.0 to +2.3 mm, reflecting the diverse deformation patterns within the study area. To further verify the reliability of the fusion results, residuals from cross-validation were statistically analyzed, and a histogram of residuals was plotted for diagnostic purposes. This approach not only confirmed the unbiased nature of Kriging interpolation but also revealed the distributional characteristics of the errors. As shown in Fig 13(b), residuals are approximately centered on zero with a near-symmetric distribution and no significant systematic bias, confirming that the errors remain within an acceptable range.

## Discussion

This study integrated the U-Net Deep learning model with time-series InSAR deformation monitoring techniques, further enhanced through Kriging interpolation, to achieve both object recognition and dynamic monitoring of Benggang in Yangkeng Town, Wuhua County. The proposed approach produced promising results. Based on the experimental findings, the discussion can be structured around the following aspects.

### Adaptability and stability of the U-Net model in Benggang recognition

This study demonstrates that the U-Net model performs exceptionally well in Benggang recognition, particularly when ResNet-50 is adopted as the backbone network. Compared with the VGG-backed U-Net, the ResNet-50-based model exhibited consistently better segmentation performance across accuracy- and overlap-oriented metrics, suggesting stronger boundary delineation and improved robustness in Benggang recognition [56]. This discrepancy primarily stems from differences in feature extraction capability and robustness between the two backbones. While the VGG architecture is relatively simple, it is prone to gradient vanishing and overfitting in deeper layers, leading to weaker boundary delineation. In contrast, ResNet-50 leverages residual connections to effectively mitigate network degradation, thereby enhancing feature representation and boundary preservation in complex terrains [56,57]. Similar findings have been reported in the literature: Meena et al. [58], in a study on landslide detection in the Himalayas, found that U-Net outperformed traditional models such as random forests and support vector machines in capturing complex boundaries from high-resolution imagery. Likewise, Guo et al. [59], through experiments on WHU, Massachusetts, and Inria building datasets, demonstrated that U-Net achieved competitive results in building footprint extraction, showing high accuracy, sharp boundary delineation, and reliable generalization across diverse urban environments.

It is worth noting, however, that during our experiments the U-Net model exhibited certain limitations in densely vegetated areas, where local omissions and blurred boundaries were observed. This indicates that conventional convolutional structures remain constrained in handling highly complex textures and multi-scale features. To address this issue, recent studies have proposed integrating Transformer architectures or multi-scale attention mechanisms to strengthen global dependency modeling and edge sensitivity. For example, Wang & Miao [60] showed that their deep residual U-Net (RU-Net), when applied to WHU and Inria aerial image datasets, significantly outperformed standard U-Net and other state-of-the-art methods in building extraction tasks, with sharper boundaries, higher IoU and F1, and much better multi-scale feature representation thanks to the use of ASPP and focal loss [60]. Future work may further explore U-Net–Transformer hybrid models to enhance the robustness of Benggang recognition and improve adaptability across diverse landscapes [61,62]. When extending to temporal neural networks for representing deformation dynamics, one may refer to the stability theory of hybrid delay and random switching neural networks to conduct robustness and stability analysis of the model [63]. From a dynamical-systems perspective, prior work on equilibrium-point neighborhoods shows that secular terms may arise under critical conditions and can be mitigated by suitable choices of initial conditions to yield periodic responses; this perspective may serve as a conceptual reference for future time-series deformation modeling and interpretation [64].

### Spatiotemporal characteristics of Benggang deformation and technical comparison

In terms of deformation monitoring, SBAS-InSAR and D-InSAR respectively demonstrate advantages in temporal continuity and spatial resolution. The SBAS-InSAR results indicate that subsidence is primarily concentrated in the exposed and intensively developed Benggang clusters in the northwestern part of the study area, showing a pronounced seasonal trend characterized by substantial subsidence during the rainy season and slight subsidence during the dry season. This trend is consistent with the findings of Yang et al. [65], who reported a lagged response of landslides to reservoir water level fluctuations, suggesting that Benggang development is strongly influenced by hydrological drivers. Further analysis of monthly precipitation sequences revealed that surface subsidence rates increased significantly during periods of heavy rainfall, whereas the deformation stabilized or even exhibited slight uplift in drier months. These observations indicate that hydrological disturbances during the rainy season may directly trigger shallow soil instability and surface subsidence.

Meanwhile, certain Benggang areas exhibited slight surface uplift. The potential causes of this phenomenon may include: (1) the rugged topography of the Benggang region, where valley wind effects promote the transport and accumulation of fine sand and topsoil; (2) abundant precipitation, which facilitates runoff-driven deposition of upstream sediments; and (3) localized construction activities and land use practices, such as soil piling, which may lead to uplift signals [66,67]. Nevertheless, subsidence remains the dominant trend across the study area, aligning with the characteristic deformation of Benggang driven by soil erosion, while other regions remain relatively stable.

**Fig 14:**
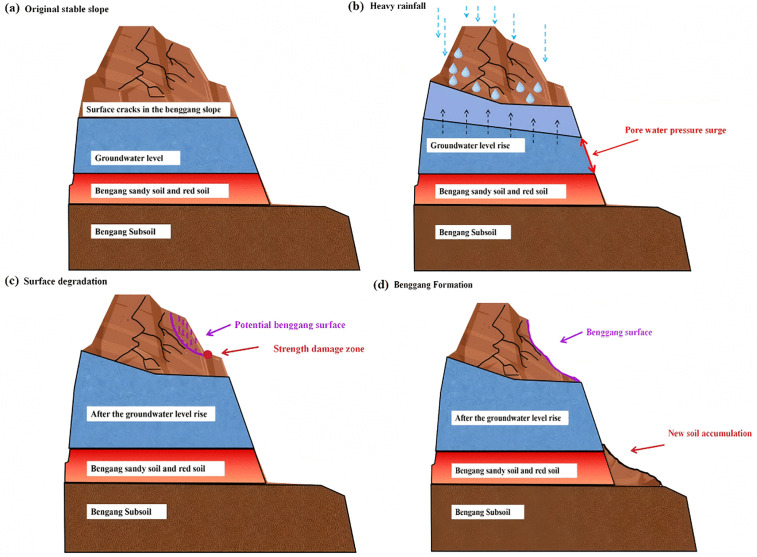
Schematic diagram of Benggang development mechanism.

Soil properties also play a pivotal role in Benggang evolution. The strata in Yangkeng Town, Wuhua County, are dominated by red soils derived from weathered granite, which are characterized by loose structure, high permeability, and weak erosion resistance [68]. Under intense rainfall, these soils are highly susceptible to erosion, leading to rapid expansion of Benggang and aggravation of local subsidence [67,69]. Based on our analysis, the formation and evolution of Benggang can be broadly divided into four stages (Fig 14). In the initial stage (Fig 14a), the slope surface remains relatively stable, showing only shallow soil cracks without significant gully development, and the landform maintains a quasi-equilibrium state. The second stage (Fig 14b) corresponds to heavy rainfall, when concentrated or prolonged precipitation raises the groundwater table, accelerates soil saturation, increases pore water pressure, and weakens soil strength. These processes trigger raindrop splash erosion and surface runoff, leading to the incision and coalescence of rills, small-scale collapses, and the emergence of initial scarps. The third stage (Fig 14c) is soil degradation, where repeated rainfall causes continuous wall collapses, deepening of channels, and simultaneous vertical and lateral erosion. Soil structures become increasingly fragmented, gullies expand rapidly, and slope toes experience intensified undercutting while slope shoulders develop cracks. In areas with loose soils or sparse vegetation, large soil blocks readily collapse, exposing potential Benggang surfaces. Finally, in the formation stage (Fig 14d), the slope structure is extensively destroyed, and active Benggang surfaces are established. Loose soils slide downslope under gravity, forming new deposits at the slope toe. At this stage, gully channels expand rapidly, scarp walls become steep, and the combined effects of headward erosion, downcutting, and lateral erosion dominate. Without vegetation cover or engineering interventions, these processes will persist under subsequent rainfall, leading to continuous expansion of Benggang and its eventual development into severe regional soil erosion units.

The monitoring results from the three sites (A, B, and C) in the study area reveal distinct seasonal deformation cycles closely associated with regional hydroclimatic conditions and geomorphological characteristics. Analysis shows that the deformation trends of all three points generally follow rainfall variations, with additional modulation by local topographic settings. Point A exhibits a seasonal deformation pattern characterized by pronounced subsidence during the wet season (April-September) and partial recovery (slight uplift) from November to March. This pattern is consistent with pore-water-pressure-driven elastic deformation: during the rainy season, prolonged infiltration raises pore-water pressures within the weathered granite regolith, reducing effective stress and promoting downslope displacement; during the subsequent dry season, pore-water pressures dissipate, effective stress recovers, and the soil skeleton undergoes partial elastic rebound. The incomplete recovery (i.e., net cumulative subsidence over time) indicates that a portion of the wet-season deformation is irreversible, reflecting progressive soil structure degradation. This mechanism is distinct from clay swelling – shrinkage behavior and does not require the presence of expansive minerals. Point B, located in a low-lying valley, occasionally shows uplift during the wet season, which can be attributed to sediment deposition transported by upstream runoff. Conversely, the subsidence observed in autumn and winter likely reflects the compaction and consolidation of deposited materials, a deformation pattern representative of similar geomorphological environments. Point C demonstrates strong subsidence during the rainy season followed by partial rebound in autumn and winter, indicating high sensitivity to hydrological variations, likely influenced by local soil–rock structure and drainage conditions. It is noteworthy that, despite seasonal oscillations at individual sites, the overall subsidence trend across the Benggang region throughout the monitoring period highlights ongoing surface deformation, providing critical evidence for regional stability assessment.

### InSAR data fusion and accuracy assessment using kriging interpolation

D-InSAR demonstrates greater sensitivity in short-term deformation monitoring, enabling the detection of surface dynamics at monthly or even shorter time scales. It is particularly effective in capturing localized deformation in small-scale areas such as slopes, roads, and farmlands. However, its results are often degraded by coherence loss and atmospheric delays, leading to noise and deviations. By contrast, SBAS-InSAR, through the construction of small baseline interferometric pairs from multi-temporal images, provides superior temporal continuity and long-term deformation tracking, though it tends to produce gaps in low-coherence regions.

To overcome these limitations, Kriging interpolation was employed to fuse the deformation fields of both techniques, thereby complementing spatial deficiencies and enhancing the continuity and interpretability of the monitoring results. Cross-validation was conducted by sequentially removing original observation points, predicting their values using the remaining dataset, and then comparing predicted values against the excluded ones. The accuracy assessment showed high consistency, with R^2^ reaching 0.978, RMSE of 0.544 mm, and MAE of 0.313 mm, confirming the scientific validity and robustness of the fusion strategy. This integrated approach is also supported by Zhu et al. [70], who demonstrated that combining D-InSAR, SBAS-InSAR, and UAV data could achieve centimeter-level monitoring accuracy, particularly suitable for complex deformation areas such as subsidence funnels. While acknowledging that internal cross-validation is not a substitute for independent external datasets, this method remains a robust alternative in geodetically sparse regions. It ensures that the fusion results are spatially coherent, even if the absolute error remains unanchored by in situ measurements. Consequently, the results are interpreted as representing relative precision [71].

The integrated “recognition–monitoring–fusion” framework proposed in this study provides a novel pathway to construct the spatiotemporal logic chain of Benggang evolution. Specifically, by superimposing deformation rate fields onto the extracted Benggang boundary layers, potential hazardous areas can be visualized directly, facilitating early identification of landslide precursors. This strategy is consistent with the integrated monitoring framework proposed by Carlà et al. [72], who demonstrated that coupling ground-based and satellite SAR observations enhances landslide mapping accuracy and strengthens early-warning capabilities in complex terrain. Likewise, Bordoni et al. [73] emphasized the effectiveness of incorporating InSAR-derived deformation fields within systematic detection frameworks (GMA-D) to delineate active ground motion areas, providing a robust pathway for large-scale geohazard surveillance. In addition, Novellino et al. [74] showed that combining InSAR time-series data with machine learning techniques enables dynamic risk assessment of slow-moving landslides, thereby facilitating the identification of hazardous zones and supporting targeted disaster prevention measures.

### Limitations

The proposed fusion framework performs optimally under sufficient SAR image density, moderate vegetation cover, and stable coherence conditions. In areas with dense vegetation, rapid land-cover changes, or prolonged temporal baselines, coherence degradation and atmospheric disturbances may reduce the robustness of deformation estimates. Sustained data acquisition, systematic coherence screening, and integration of complementary observations are recommended for operational deployment.

It should be noted that Points A, B, and C are representative monitoring locations selected to illustrate deformation characteristics at distinct geomorphological positions, not geodetic reference benchmarks. The InSAR reference point anchoring all deformation measurements is a separate stable location outside the active erosion zone, as described in the Methods section.

Independent ground-truth validation using geodetic instruments (e.g., GNSS receivers, leveling benchmarks, or corner reflectors) was not available for this study. All reported deformation values represent relative line-of-sight displacements referenced to the stable point above, and absolute accuracy verification therefore remains a limitation. Future work should incorporate permanent GNSS stations or corner reflectors to provide independent validation and convert relative measurements into an absolute geodetic reference frame.

## Conclusions

This study developed an integrated stereoscopic monitoring framework combining Deep learning and multi-temporal InSAR to achieve coordinated observation of Benggang areal extent and vertical deformation. The U-Net-ResNet50 model applied to Gaofen-2 imagery demonstrated superior performance in automated Benggang extraction (IoU = 86.00%), confirming the advantage of Deep learning methods for identifying complex erosion landforms. SBAS-InSAR analysis revealed pronounced spatial heterogeneity in surface deformation during 2019–2021, with cumulative deformation ranging from −33 mm to +34 mm. Subsidence exceeding −20 mm showed strong correlation with hydrological processes. Kriging-based fusion of the D-InSAR and SBAS-InSAR results generated a spatially continuous deformation field with high internal consistency. The derived three-year cumulative deformation ranged from −14 mm to +18 mm, and internal cross-validation indicated high spatial coherence (R^2^ = 0.978), though absolute accuracy remains to be verified by independent geodetic measurements. This approach effectively compensated for monitoring gaps in low-coherence areas inherent to individual InSAR methods. The proposed framework provides a reliable technical pathway for three-dimensional dynamic monitoring of Benggang evolution and holds significant potential for early identification of geohazards and ecological risk management.
